# Medicines of Uncertainty and Objects of Care: Creative Engagement with an Ancient ‘Folding Almanac’

**DOI:** 10.1007/s10912-025-09954-5

**Published:** 2025-05-30

**Authors:** Sarah Scaife

**Affiliations:** 1 University of Exeter, Exeter, United Kingdom; 2 University of Bristol, Bristol, United Kingdom

**Keywords:** Uncertainty, Cancer, Making, Holding, Relationship, Ritual

## Abstract

Folding almanacs are magico-medical objects which were worn and used by doctors in fifteenth-century England to perform rituals of medicine and to align the timing of diagnosis, prognosis and treatment to earthly and cosmic cycles. As a multimedia artist, my curiosity was taken by these hand-held objects of care. To my contemporary eye, they are essentially artist books. A further connection came through my own lived experience of breast cancer. A year of intense treatment, including six cycles of chemotherapy followed by mastectomy, significantly complicated my relationship to my own body and to medicine. This creative engagement explores how and why I tried making my own folding almanacs, using modern materials, and what I learned when one of these was accepted for *Un-boxing*, an international travelling exhibition. These ancient folding almanacs encapsulate a world view where people’s lived experiences of being in a body was held within a flow of relationships with other bodies, human and non-human including animals, the moon, stars and planets. A close reading of the visual and material languages I used in this remaking offers insights into a personal health history folded into bigger questions of what we might allow into an expanded field of ‘medicine’.

Folding almanacs are artefacts from fifteenth-century England, which were worn and used by doctors (Carey 2004, 2003). Similar in size to a current-day mobile phone, a folding almanac was attached by a chord to a doctor’s girdle. Its folded pages contain ‘data that enabled medical practitioners and others to diagnose and prognosticate’.^1^ In the medieval medical paradigm, astrology was one significant source of information during such processes. These beautifully made, luxury objects of care hold narrative and embody specialist magico-medical^2^ knowledge from 600 years ago. However, ‘magico-medical’ is a contemporary label. I refer to ‘magic’ here not as conjuring or superstition, but rather as discussed (in a different context) by Kotva (2017). Any magical agency accessed through these objects is derived from their use in a performance of healing which takes place embedded in a set of relationships beyond those typically made explicit in secular, scientific, evidence-based medicine (though explored in the medical humanities discourse). This magic is relational and involves paying close attention to the environment, including the cosmos. Unfolded alongside the patient in the sick room, these objects also embody rituals and power dynamics of doctor-patient relationships (Bolaki 2016) as they were performed in that period and context. Thus, such almanacs, both historically and of my own making, implicate a medicine enfolded in well-studied and yet still wondrous forces beyond the zone of reason (Fig. 1).

**Fig. 1:**
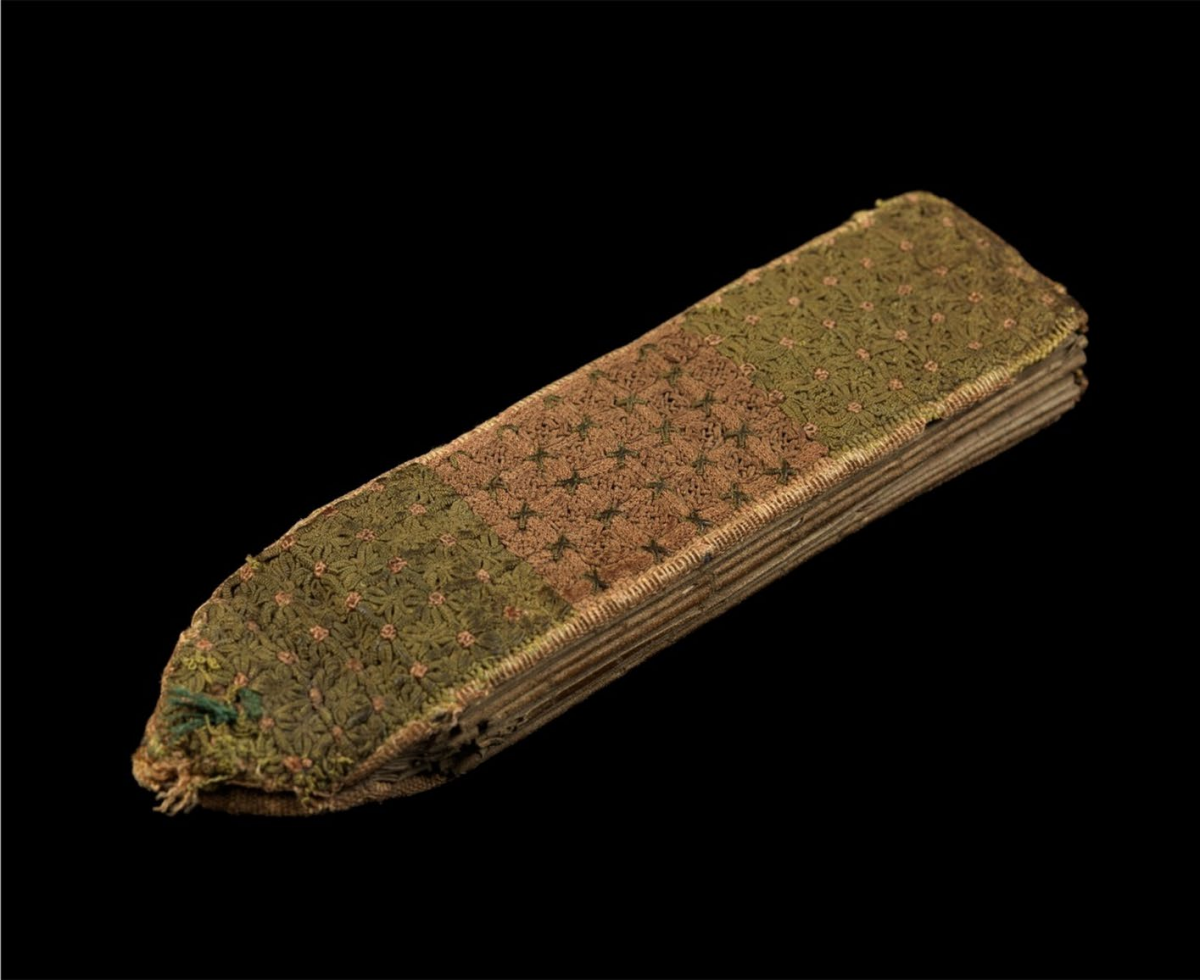
English folding almanac in Latin, c. 1415–1420. Embroidered textile binding is now a faded green and pink. Object size 38 mm × 160 mm. Public Domain Mark. Source: Wellcome Collection (MS.8932) https://wellcomecollection.org/works/a2y8zd6x

This creative piece focuses on folding almanacs as objects of care. As a multimedia artist, my curiosity was taken by these hand-held objects of care. To my contemporary eye, medieval folding almanacs which survive are essentially handmade artist books. A further connection came through my own lived experience of treatment for breast cancer. At an early stage of my PhD enquiry, I set out to learn more. This meant viewing folding almanacs online (particularly Wellcome MS.8932) and experimenting by making a few folding almanacs myself using modern materials. Later, I found an unexpected opportunity to share an almanac in an international travelling exhibition (Figs. 2 and 3).

**Fig. 2:**
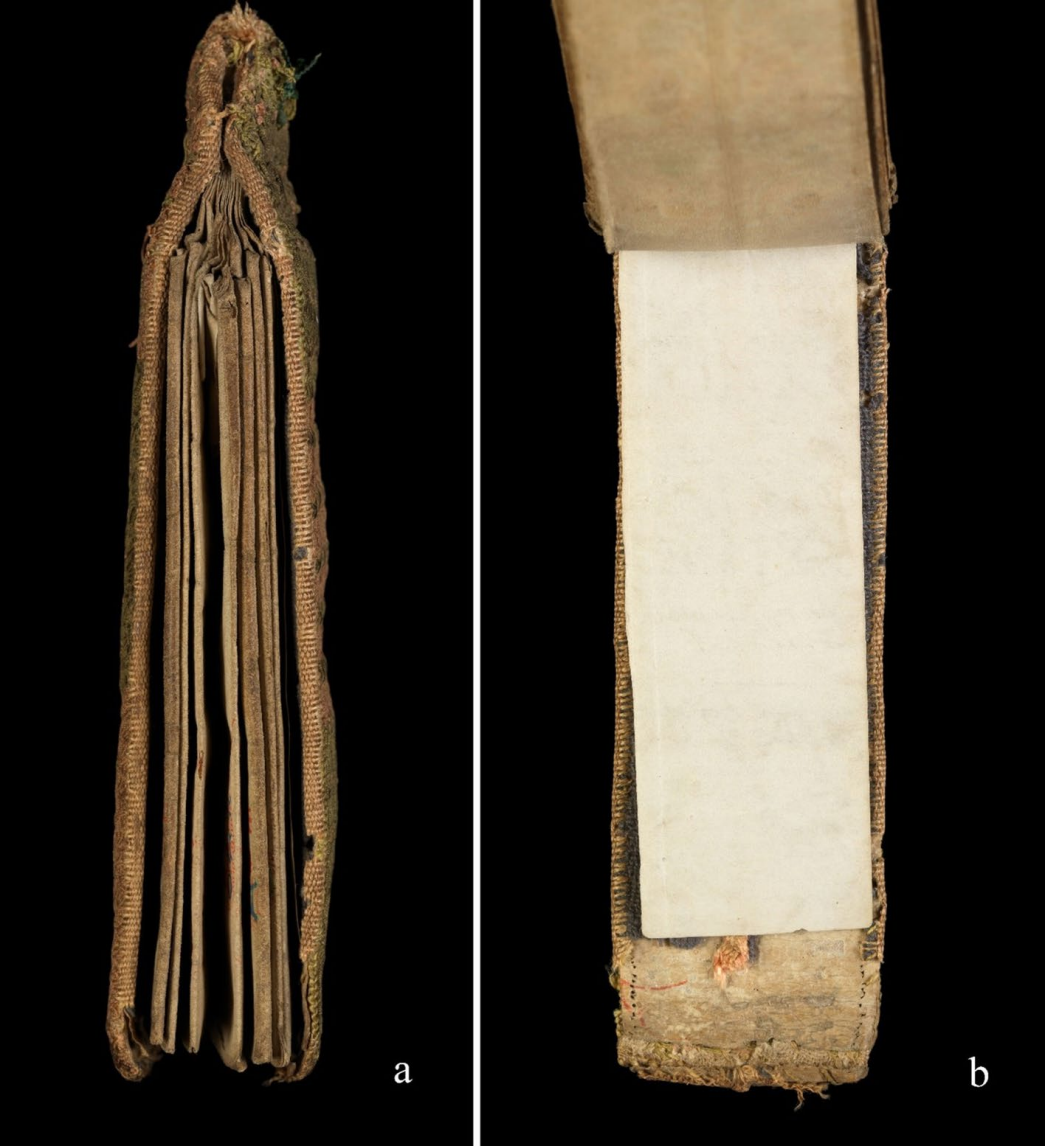
English folding almanac in Latin, from sideways on (**a**) and opened up (**b**). Public Domain Mark. Source: Wellcome Collection (MS.8932)

**Fig. 3:**
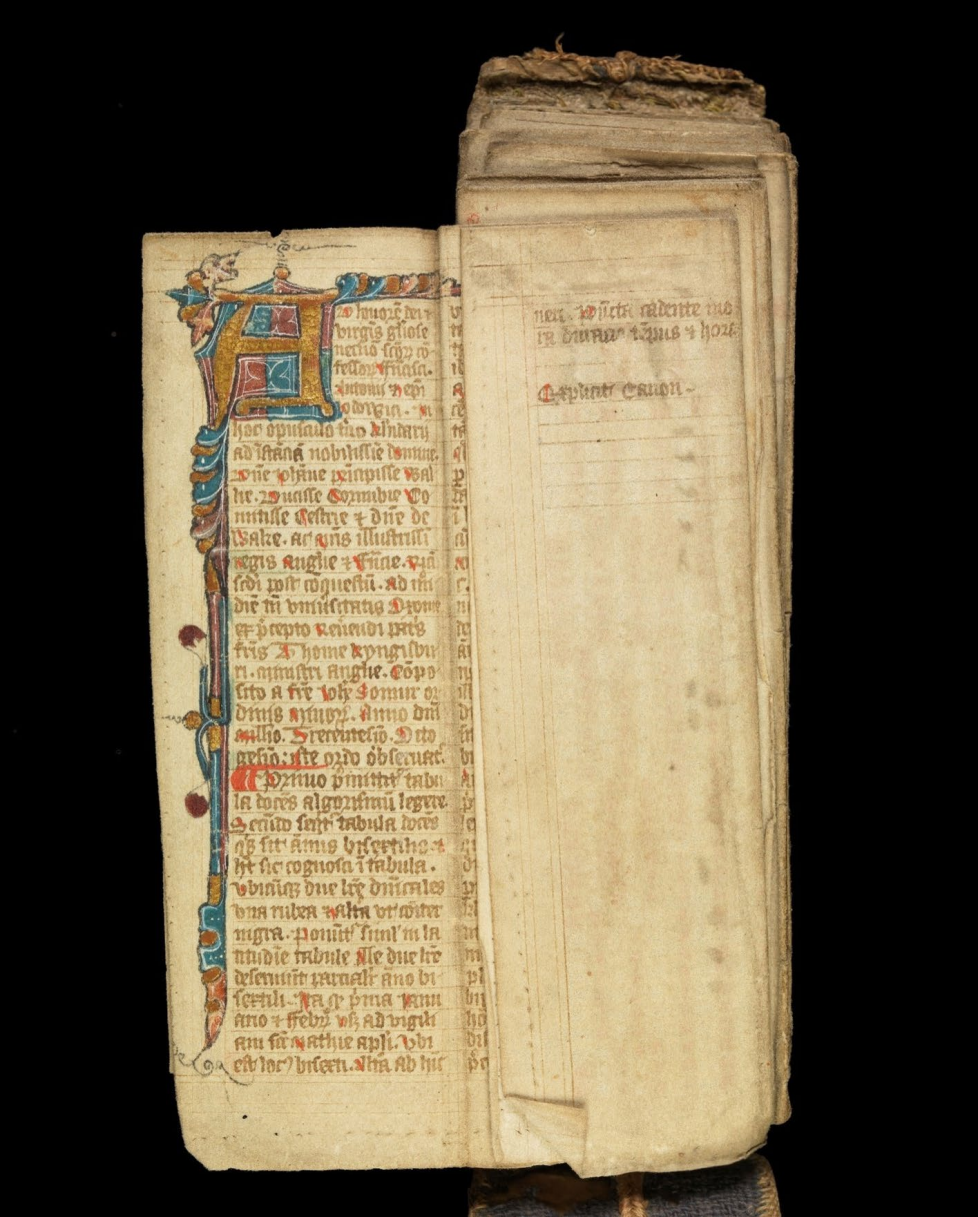
English folding almanac in Latin, with leaves partly unfolded to reveal handwritten and illustrated contents. Public Domain Mark. Source: Wellcome Collection (MS.8932)

This PhD research emerged out of my lived experience of breast cancer treatment (from 2017). A year of intense treatment, including six cycles of chemotherapy followed by mastectomy, significantly complicated my relationship to my own body and to medicine. Self-care around such a challenging and emotional patient experience is a long-term process of acceptance. One of the things I drew on was my existing creative practices and interests. As a research artist, engaged in a Master’s degree at the time of diagnosis, I found that my critical and creative tools and making skills could help to articulate, illuminate, and process such unanticipated life events.

## Practice-based research in creative health

As an artist and postgraduate researcher at the University of Exeter, UK, I explore ‘medicines of uncertainty’ in a more-than-human world. My PhD enquiry uses a practice-based research methodology, which models tacit, embodied and multifaceted ways of (not) knowing. Biomedical research develops targeted pharmaceutical treatments for named conditions, but cross-culturally and in my lived experience, medicine is more than this. This paper investigates what might be the expanded scope of medicine at the confluence of health, environment, ecology, and the arts.

## Folding together more-than-human relations

**Fig. 4:**
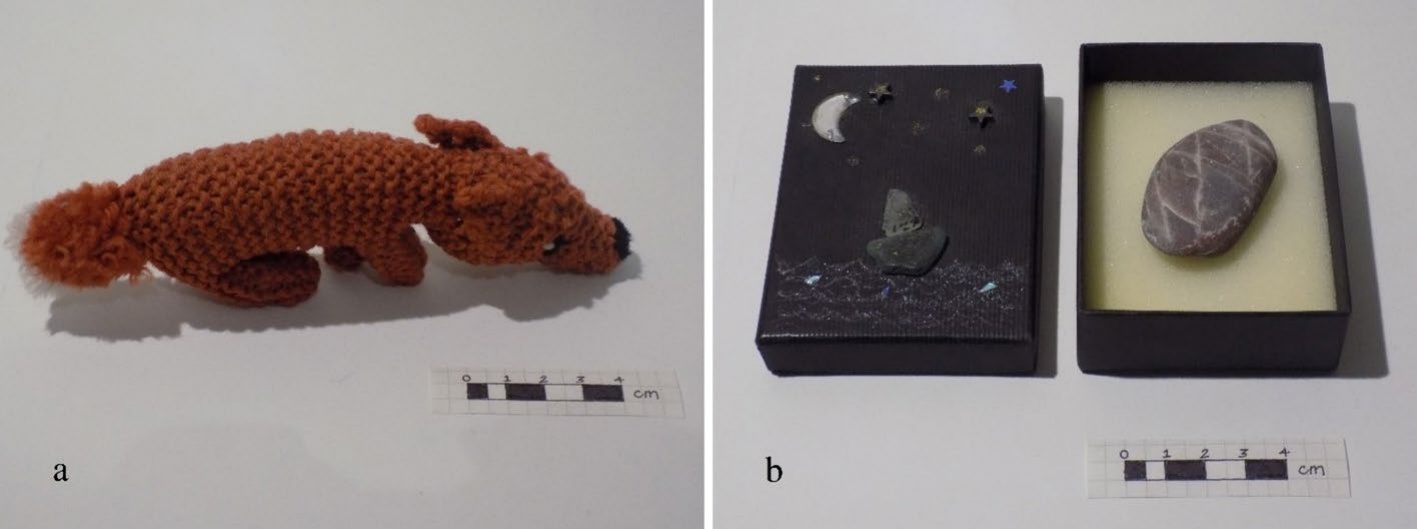
Examples of objects of care in the author’s personal collection: hand-knitted fox (**a**); beach pebble in a decorated gift box showing moon and stars over the sea (**b**)

I am curious about small, portable objects which can be carried in the hand or about the body and implicate relational, ritual practices within healthcare. During cancer treatment, I curated and used a personal collection of dozens of such objects of care, typically selecting a few to wear as amulets and talismans, or to carry to hospital appointments hidden in my pockets. My collection includes site-specific pebbles and fossils, animal figures which embody particular qualities, and particular pieces of jewellery such as a snake-like necklace which I regularly wore to meet the venom of chemotherapy. Some objects in this collection are precious gifts from loved ones, some I came upon during intentional walking,^3^ others I made myself. Some of these personal objects of care are reminiscent of archaic artefacts including prehistoric so-called ‘Venus’ figurines, and votive body parts (see for example, Hughes 2021; Meskell 2017).^4^ (Fig. 4).

**Fig. 5:**
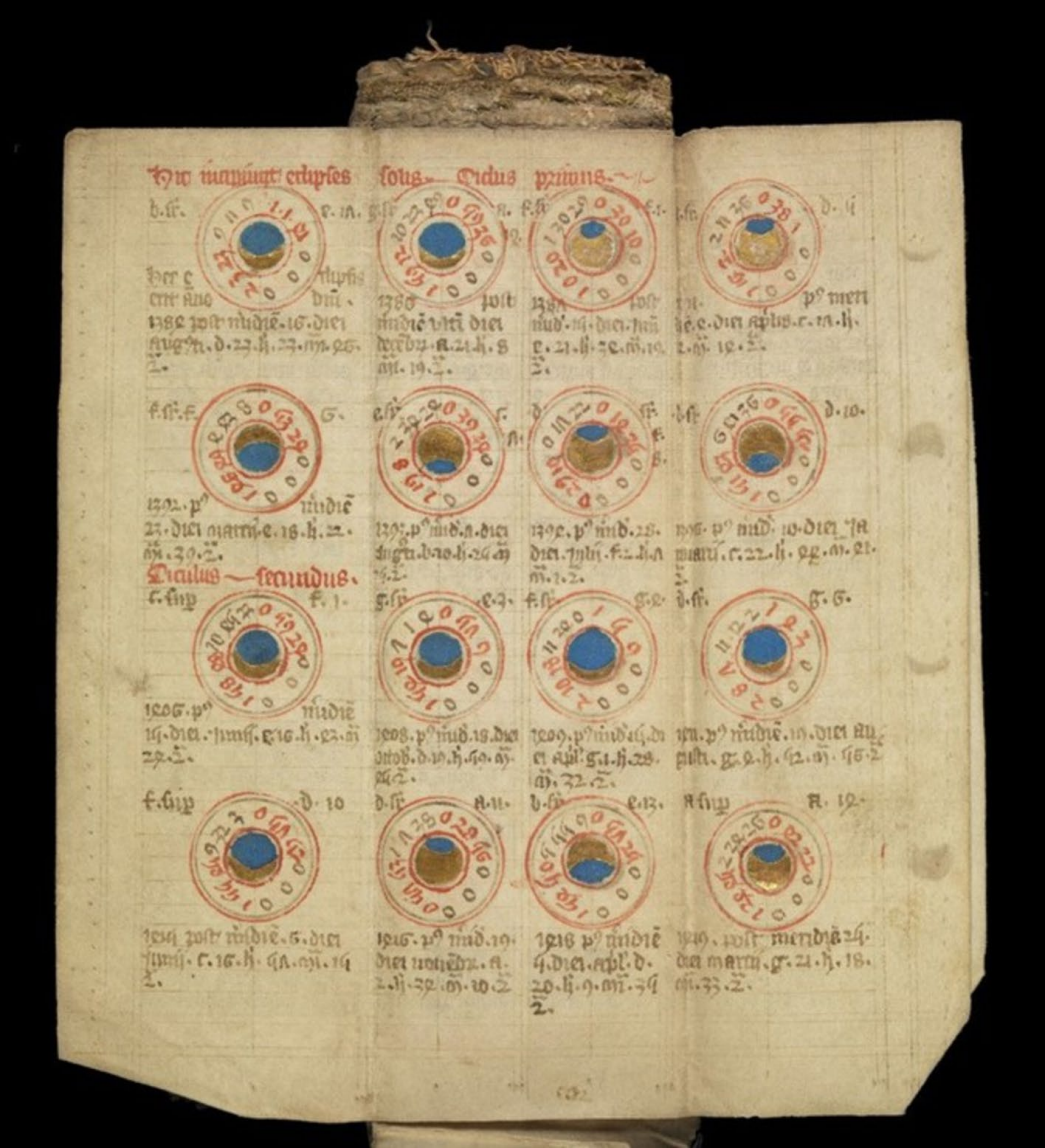
English folding almanac in Latin, unfolded to show cosmic cycles. Leaves when unfolded measure 106 × 236 mm. Public Domain Mark. Source: Wellcome Collection (Wellcome Library MS.8932)

**Fig. 6:**
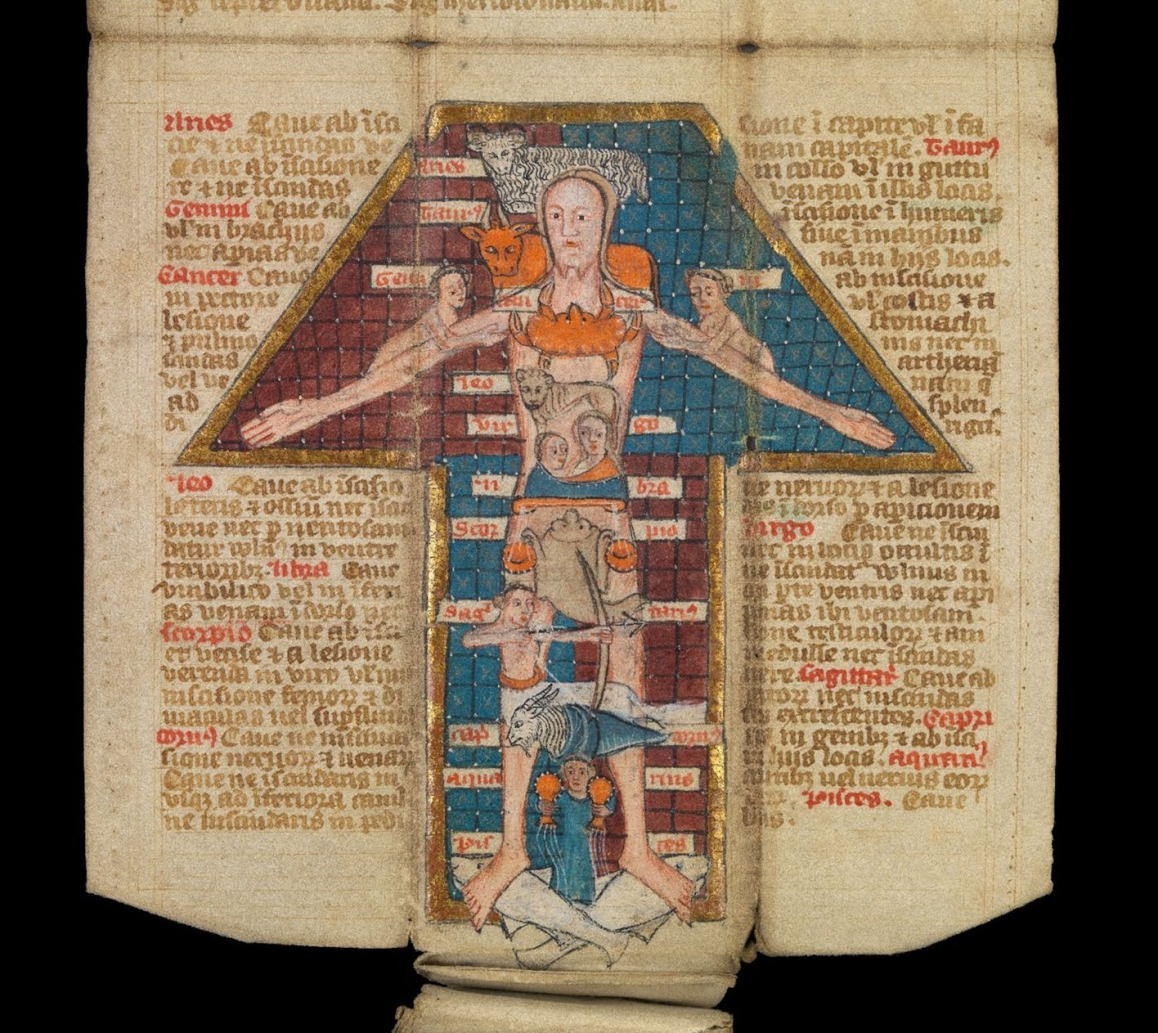
English folding almanac in Latin, unfolded to show a Zodiac Man in blue, red, dark red and brown ink with gilt border. Public Domain Mark. Source: Wellcome Collection (MS.8932)

Folding almanacs are among several classes of objects of care which particularly interest me. Through engaging with folding almanacs, I continue to expand my sense of what counts as medicine. In this creative engagement, I learned that medieval physicians debated the extent to which ‘causes distant from the body’ of a patient needed to be taken into consideration for effective treatments. Astrological tables and diagrams folded into the almanacs indicate relationships considered significant to health at that time (Figs. 5 and 6). For example, calendars within these almanacs typically indicate the position of the sun, the phase of the moon, holy days and related auspicious times for bloodletting (Carey 2004, 350).

Cultural assumptions, stories and beliefs can be jolted through experience of disease. In a flood of uncertainty, we might clutch at what we know or feel that the ground beneath us is slipping away. A family friend lost her lifelong religious faith as a result of the shock of a cancer diagnosis. Whilst my own cancer treatment sucked me into a cyclone of scientific medicine, I rejected narratives of fighting the enemy within and expanded my sense of the benefits of cyclical creative practice in community and in nature local to where I live. Though I deeply appreciate the biomedical technologies and expertise which saved my life, I also sought antidotes to the intensity of oncological data, predicted outcomes, and high-tech indoor environments. During almost a year of treatment, I repeatedly walked along a tidal stretch of river close to my home, often with my spouse or a friend, sometimes at dusk alert to the rising moon. Illness enlivened my existing ritual connection with more-than-human ‘nature’ and I feel an affinity with illustrations in the medieval almanac, even though I am from another time (Bolaki 2018; Gilchrist 2020).

This lived experience underpins a second strand of my practice-research: intentional walking. I made a series of radio programmes to explore the efficacy of intentional walking as a connective practice, and this in turn informed a collaborative series of walking-based research gatherings with people who have experienced breast cancer treatment (Scaife 2024, 2021a, 2021c, 2020). As we sauntered together, natural objects and sometimes animals presented themselves to our imagination (Scaife 2021c). On one very wet day, our walking itself took place in our imagination, as I led a guided visualisation. We held these freely-given objects—such as a feather, a leaf skeleton or a seed pod—and found that they supported us to speak of our experiences and hopes.

**Fig. 7:**
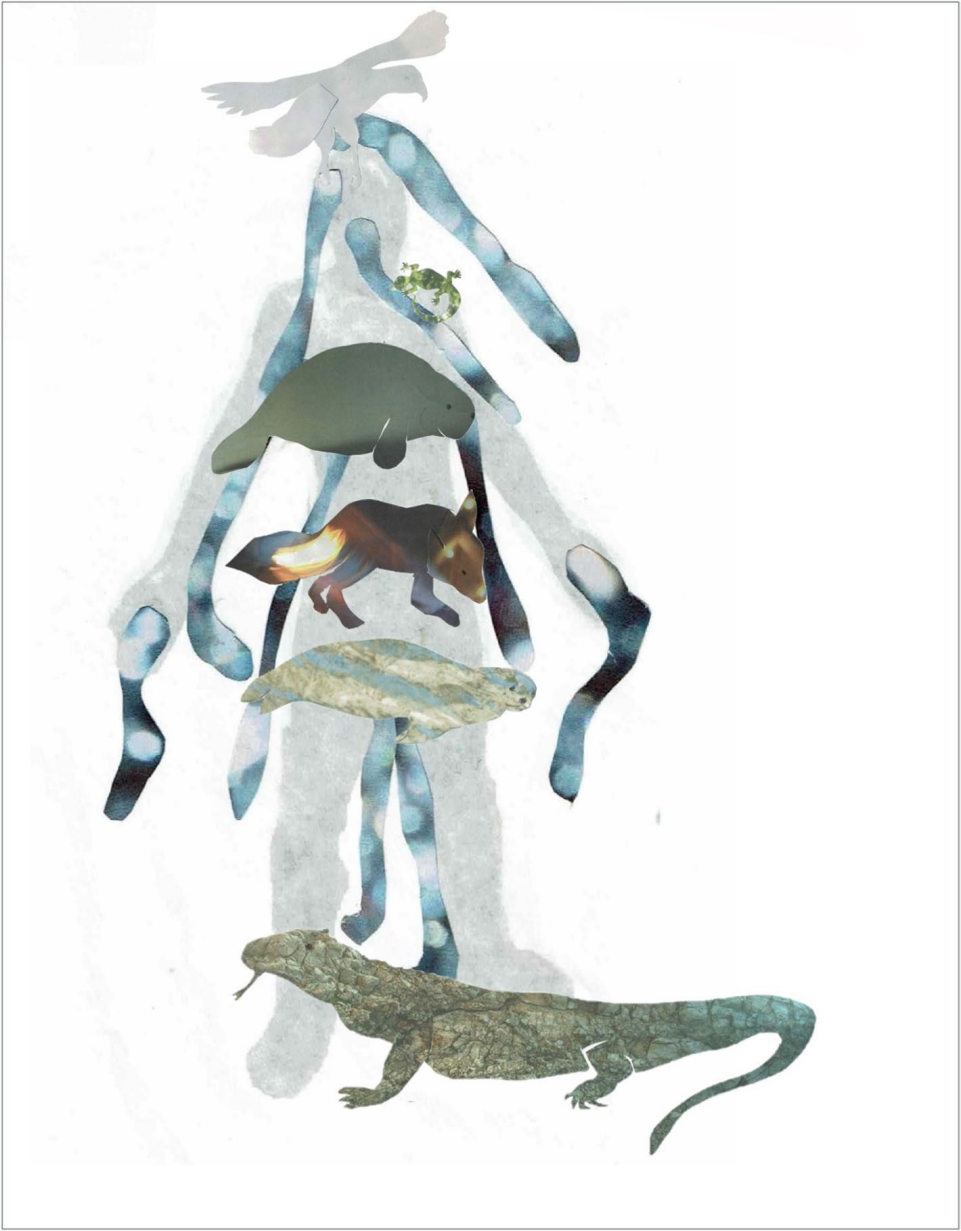
Sarah Scaife, self-portrait with animals after Zodiac Man in folding almanac Wellcome MS.8932. Collage on paper

In my personal practice, I have a long-standing relationship with the inner world of the imaginal and regularly work with the dream-like qualities of a site or animal archetype. Intrigued by the medieval body map of the ‘Zodiac Man’ included in the pages of folding almanac MS.8932 (Fig. 6), I revisited an earlier reflection on the cosmological stories held in my own physical body (Scaife 2021b). This led to a self-portrait which locates personal connections to animal archetypes within my body (Fig. 7). Here, rather than the crab of Cancer in the Zodiac Man, a dugong floats across my chest: these endangered, gentle sea mammals live in coastal-water, responding to tidal fluctuations. Like human women, dugongs have two mammary glands towards the head of their belly. You may also notice Fox, an archetypal representation of resourcefulness and adaptability, one of the animals in my objects of care collection (Fig. 4).

## Being in a body held within a flow of relationships

These ancient folding almanacs encapsulate a world view where people’s lived experiences of being in a body was held within a flow of relationships with other bodies, human and non-human including animals, the moon, stars and planets. It is easy to view the relational charts and images in these old objects as quackery. Why would the twenty-first century Western medicine take account of cosmic cycles? But studying and re-making folding almanacs also confirmed my sympathy for some of the underpinnings of medieval medicine and care. In my own health experience, I feel a close connection with the need to attend to the bigger cosmos and natural cycles. In a time of deep uncertainty, such ebbs and flows and such changes of scale bring comfort and perspective (Scaife 2021a, b, c; Scaife 2020).

Furthermore, these precious folding almanacs can be read in more ways than just their handwritten and drawn contents. Consulting digital images freely accessible online through the Wellcome Library, I set out to recreate a contemporary artistic representation of these book-like objects. In attempting to remake such an object, I was revisiting many layers of my health experience. Bolaki (2016, 2018) suggests that the material book, with its spine and other haptic attributes, can stand in for the human body. For McNamee (2020) the folded leaves within such almanacs obscure some surfaces and reveal others, in the same way that things which might seem to be unconnected can rub up and press against each other in the enfolding of the life course. Ingold (2021) asserts that a physical book is both a literal and an ideational gathering, a binding together of *correspondences*.

**Fig. 8:**
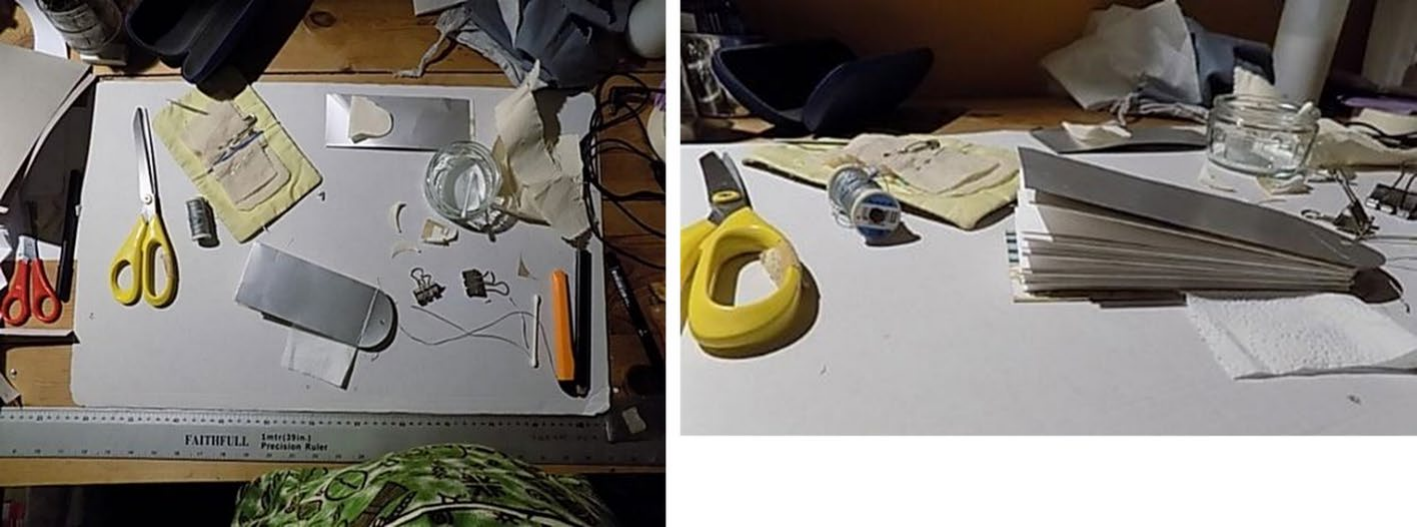
Work in progress: photos taken by the author to document making the folding almanac

**Fig. 9:**
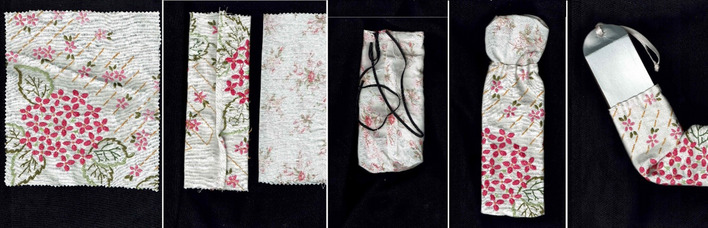
Work in progress: photos taken by the author to document making the cover of a folding almanac

From my Bauhaus-style Art Foundation, I have a basic training in traditional bookbinding. This involves stitching, folding and cutting. As I used knives, needles, threads and folding, I was aware of replicating surgical procedures experienced by my own body. The hand-made, gathered binding process took time and attention, literally stitching care into the piece. Rather than vellum, I recreated the cover from a recycled oat milk carton. This nod to calfskin vellum also speaks to the way that I stopped drinking cow’s milk after losing a breast, now preferring to leave milk to the calves. Being immersed in the processes through my hands opened a reflective time and space to think, feel, and come to terms with my need for continuing self-care (Figs. 8 and 9).

**Fig. 10:**
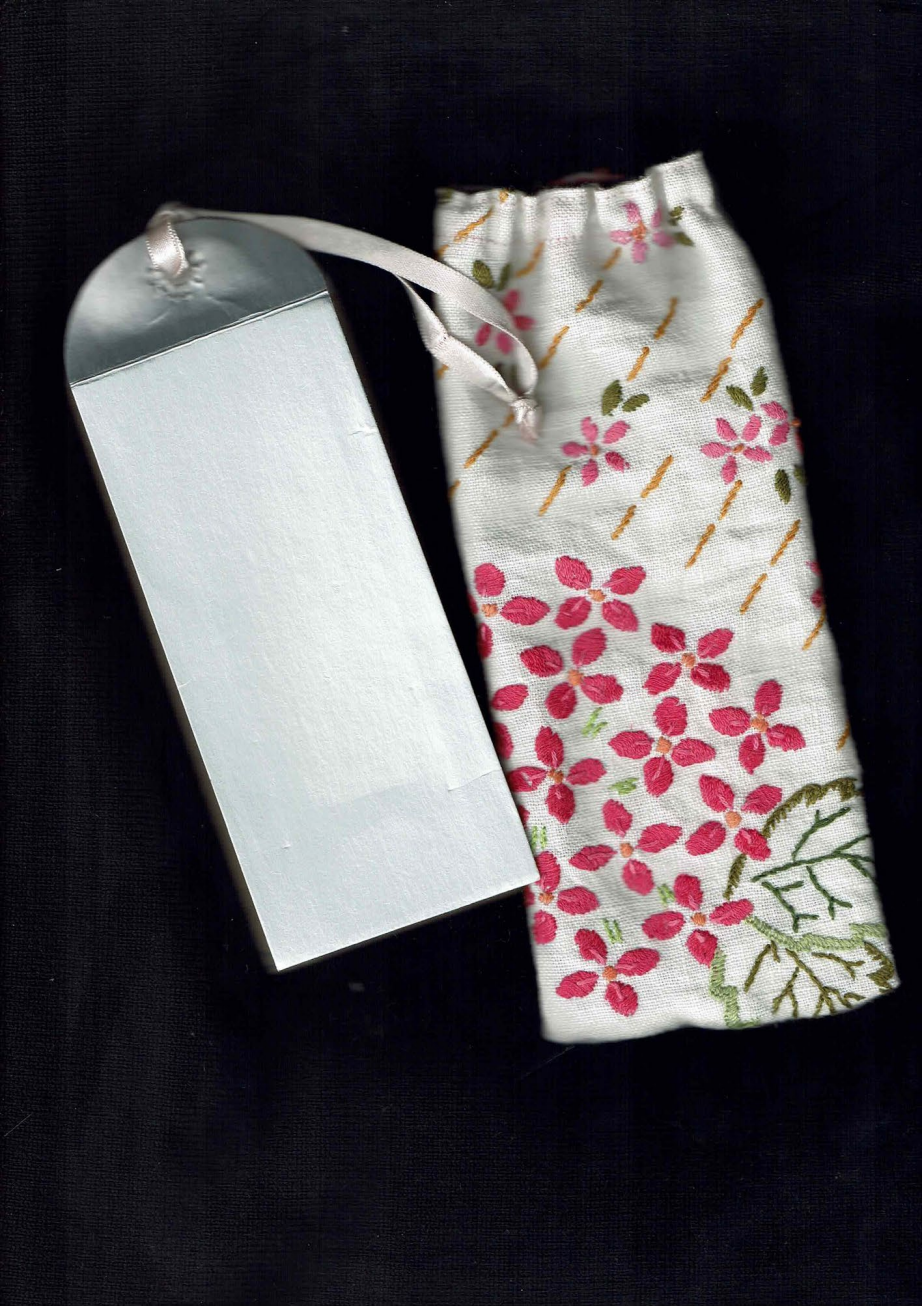
Sarah Scaife, folding almanac with repurposed green and pink textile cover embroidered in the 1960s by Constance Scaife, 2021a, b, c. Binding measures approx. 38 × 160 mm

The cover of folding almanac MS.8932 is embroidered in a pink and green floral design. My completed almanac is protected by a fabric case. I stitched this bag from the reanimated remains of a tablecloth, also a pink and green floral design, hand embroidered by my grandmother, Constance Scaife (1907–2010). She devoted herself to caring for many people, serving as a servant, a nurse, and an affectionate family member. My grandmother, despite limited opportunities, also exemplified exceptional craft skills. She lived a long life of caring and craftsmanship. This fabric fragment is thus charged with gendered notions of ancestral skill, service and survival through times of challenge. This little, embroidered bag invokes longevity, adds attractive colour and tactility and protects my newly created object (Figs. 8 and 10).

### *Un-boxing*—A travelling exhibition of care in a box

This enquiry into folding almanacs happened in 2021. *Un-Boxing*, a group exhibition in a box initiated and curated by Arts Territory Exchange (aTE),^5^ became an unexpected opportunity to share my enquiry. My contemporary almanac was accepted to join *Un-Boxing*. The show was displayed to small audiences in domestic and alternative locations in England, Wales, France, Denmark, USA and Canada for considerably longer than a year. At a time of global separation, the postal journey of the show linked artists and communities. My folding almanac became one object in a box full of objects of care connecting up isolated artists and their local supporters in an international network. This aTE collaboration was documented on their website and social media pages as it travelled, generating data in the public domain and shared in common by all the participating artists and curators.

From its making to this international journey, the folding almanac embodied and enfolded moments of health, care and connection across past and present times of global health uncertainty. At the start of the *Un-Boxing* tour, the folded pages of the almanac I exhibited were blank, except for a brief, handwritten introduction and invitation to contribute by marking the page(s). My idea was to send this almanac into the world offering the potential to develop a shared book-object of care co-created by those who might feel curious when they encountered it.

**Fig. 11:**
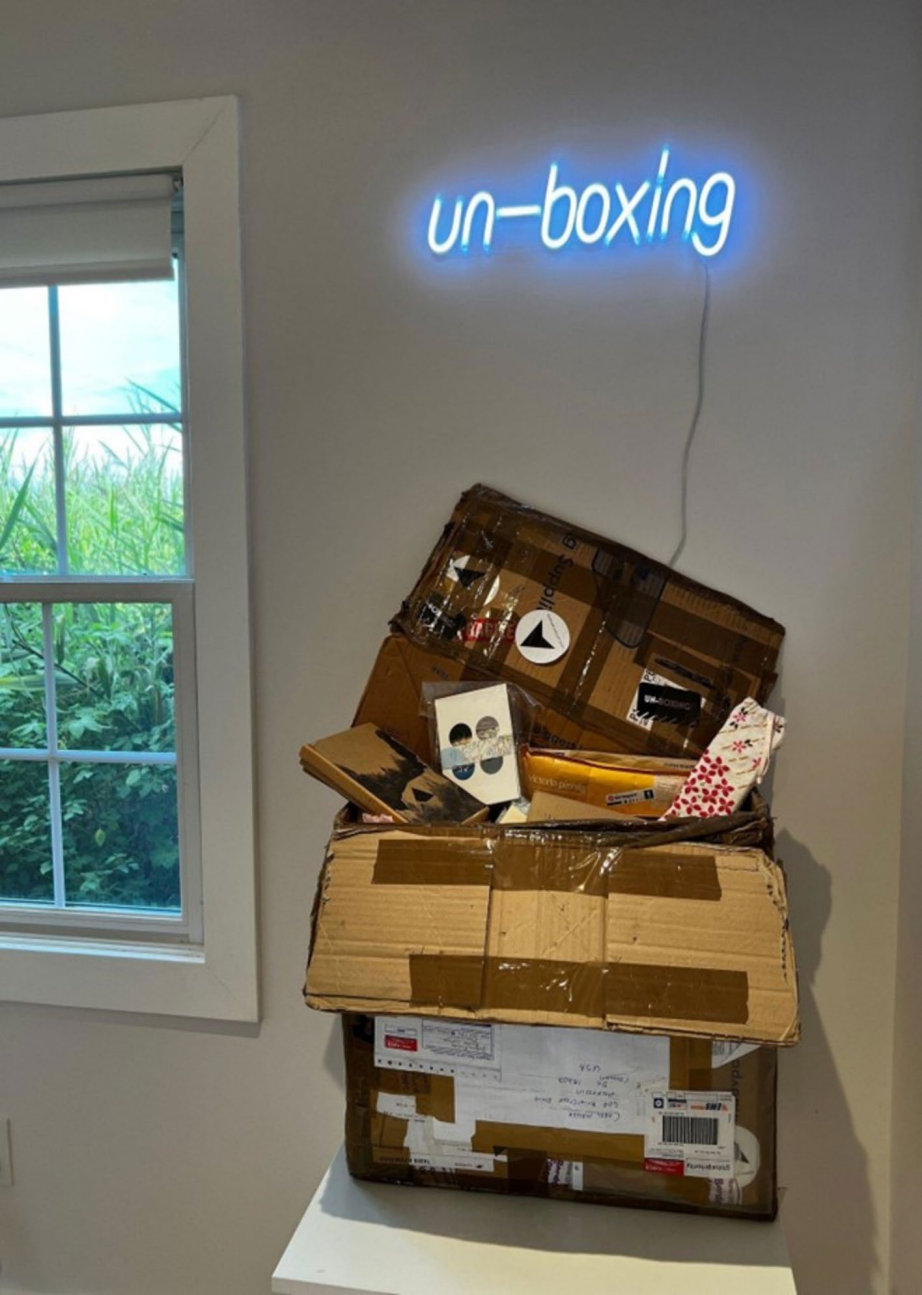
The contents of the *Un-Boxing* parcel as documented on arrival at Street Road Artists’ Space, Philadelphia, USA, where it was unpacked by visitors from 19 Aug to 19 Nov 2022. The author’s folding almanac, in its embroidered cover, is visible in the right side of the box. https://www.streetroad.org/unboxing-at-street-road.html

This handwritten invitation page (Fig. 11b) reads:

Folding almanacs such as this were first made 600 years ago, in England. They make a link between human health and lunar and solar cycles, between body, earth and sky.

Here we are spinning and flying through the universe. And at the same time we can connect to the centre of the earth and find a stillness in our own centre.

You are invited to add to this almanac.

How and where do you find connection in these times of global health uncertainty?

**Fig. 12:**
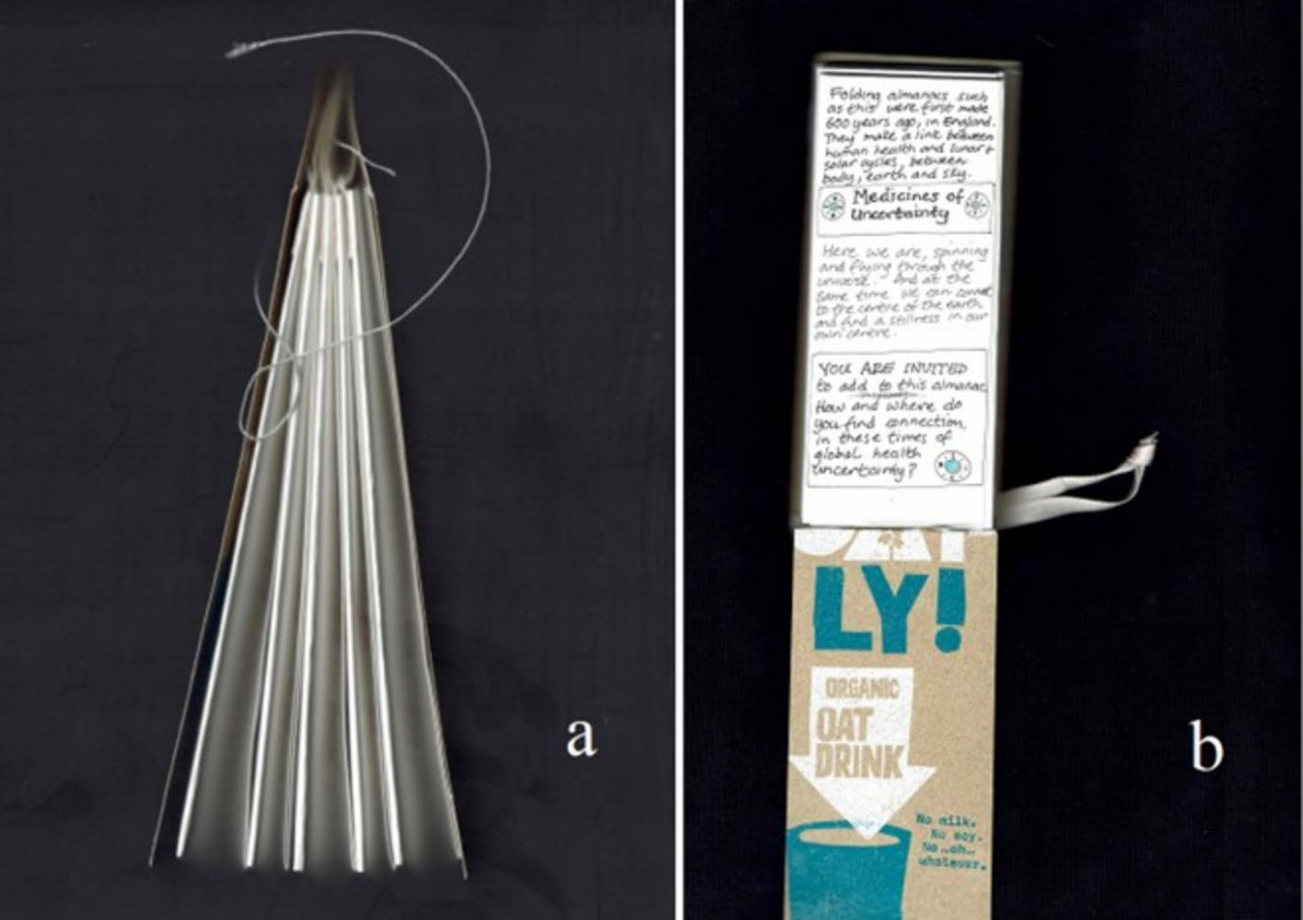
The author’s folding almanac from side (**a**) and invitation to add to the blank pages (**b**)

**Fig. 13:**
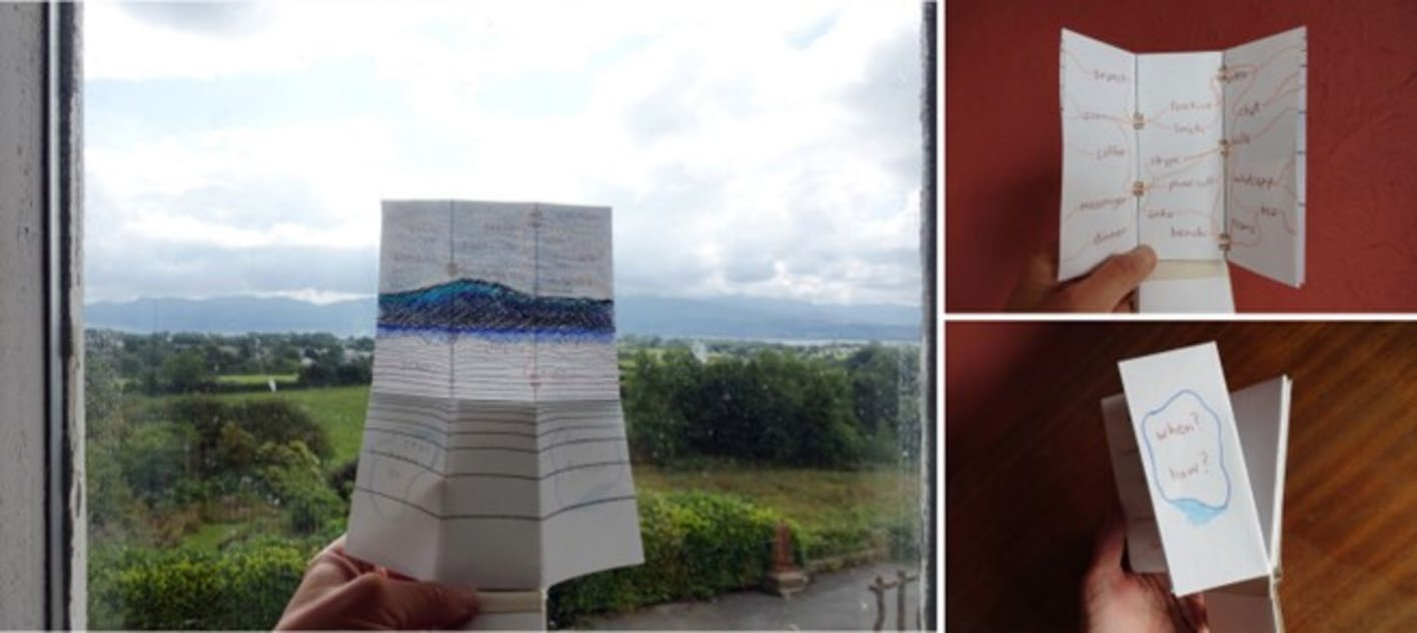
Content added at Plas Bodfa, Wales, UK, 2021 https://www.plasbodfa.com/unboxing/sarah-scaif (sic) Accessed 19 February 2025

**Fig.14:**
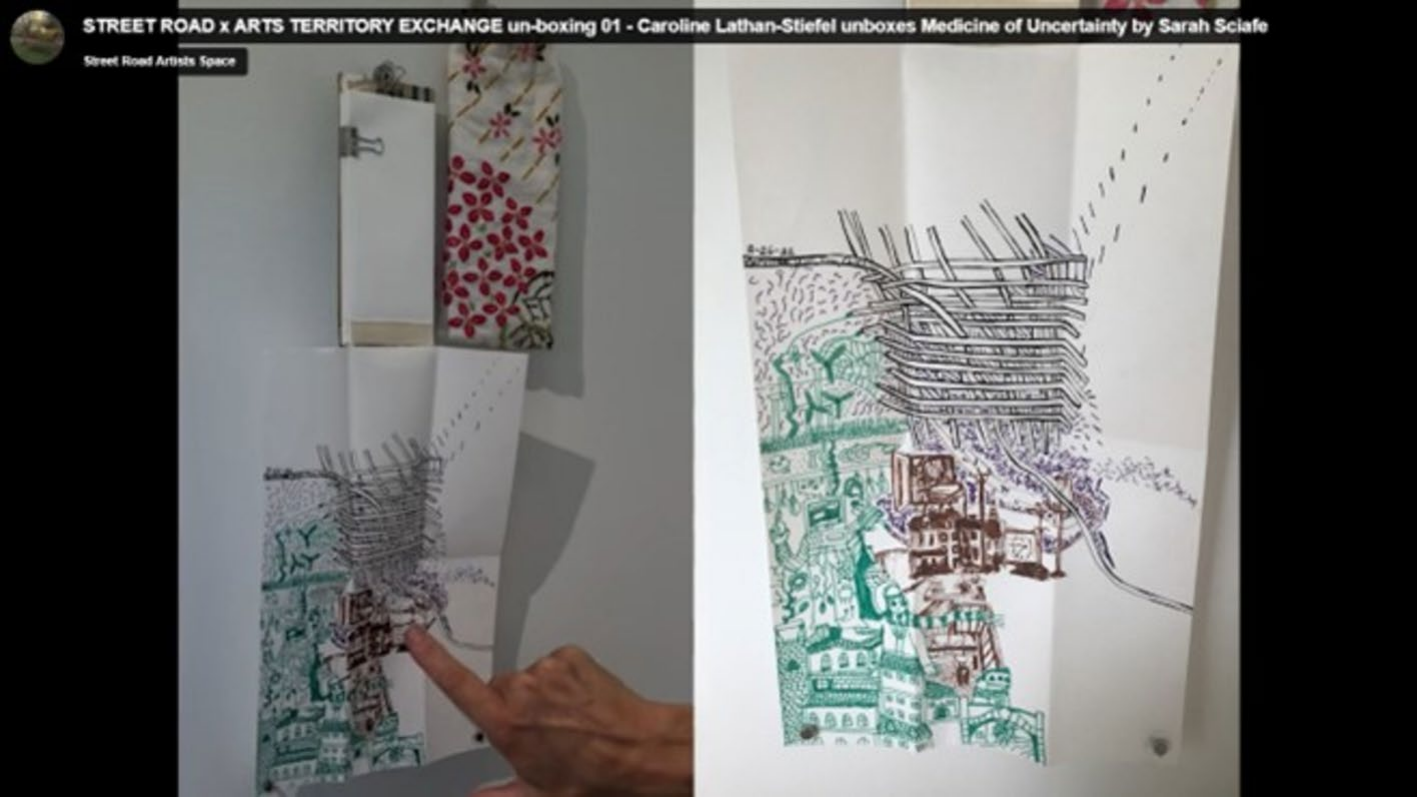
Folding almanac as displayed at Street Road Artists’ Space, Philadelphia, USA. Created by Sarah Scaife, 2021a, b, c, with drawn contribution by Caroline Lathan-Steifel, 2022 https://www.streetroad.org/unboxing-at-street-road.html Accessed 19 February 2025

Over the coming months, I watched online as the other artists and visitors posted documentation of the travelling exhibition (e.g. Figure 11, 12, 13, and 14). Street Road Artists’ Space, Philadelphia, USA, posted a video which I enjoyed watching. This five-acre site is ‘a laboratory for the consideration of humans’ multiplicitous relationships with land – past, current and future’ (https://www.streetroad.org/).

Here the audience were invited to unbox the show, piece by piece, and co-install it in the gallery. The video records artist Caroline Lathan-Steifel unboxing the almanac and responding to my invitation. With one of her children, she added a beautiful and care-filled drawing that offers a window into their world far away across the planet from me. When it is 8 pm at night in the UK and I am gazing at the moon, it is 3 pm on a sunny afternoon for her. I can see from this drawing that we both care about keeping connection with natural cycles, just as health systems did in the medieval times of the original almanacs.

One unusual condition of inclusion in the *Un-Boxing* travelling exhibition was that artists relinquished their right to the object of their making and agreed that it would not be returned. The curated collection was posted across an evolving aTE network, anchored around specific artist spaces. Its online trace fizzled out in late 2023.

In early 2025, I came across a pertinent and apparently true story told by a respected medical historian (Singer 1958, 136). It concerns Sir John Holt, Lord Chief Justice of England from 1689 to 1710. As a young man, privileged, cheeky and adventurous, Holt used his education to his own ends. Hearing that a landlady’s daughter had a fever, he scribbled some words of Greek onto a leftover piece of parchment and rolled it tightly. He exchanged this text for lodging by telling his landlady it was a charm. The rolled text was duly tied to the wrist of the girl. He stayed at her inn for a week, free of charge and the girl recovered from her fever. Many years later an old woman was brought before the same John Holt, charged with witchcraft. The key evidence against the old woman was a rolled-up scrap of parchment, described as a charm which could cure fever. Courageously Holt, in contrast to the misogyny of his time, recognised the very parchment which he had himself rolled up as a young man; he revealed this and the woman was acquitted.

When we make magico-medical objects, whether as objects of care or objects of ruse, and release them into the world, often we do not know what will become of them. Within the landlady’s presumably rather less-advantaged community, this scrap of parchment—once proven effective for the girl—had become medicine. My almanac was created in good faith as an object of care and a medicine of social connectedness. Maybe I will meet the almanac again in my own old age; maybe, like the medieval folding almanac which inspired it, unknown to me, my work will end up in an archive; or maybe it will fall apart, be recycled or end up as litter. There is some creative pleasure to be had in this open-ended uncertainty.

## Conclusion: Objects of care as holders for re-imagined body-story

In my current practice-based research, I explore how the making of artworks, ranging from magico-medical objects to intentional walking ceremonies, has the potential to generate novel kinds of eco-socio-cultural medicine. The narrative medicine strand of my PhD enquiry seeks to change the stories we tell ourselves and each other about health and who or what claims the agency in these stories. At a time when uncertain presences increasingly hold sway, making or finding objects of care to embody qualities or re-imagine elements of body-story became more present in my research practice. What the objects of care explored here have in common relates to holding: they hold story and with this a certain amount of withheld, esoteric resonance; along with a physical scale relating to the human body, often just right to be held in a hand. The remaking of ancient almanacs offered opportunities to develop and claim metaphors of care for myself and then offer these to others. A close reading of the visual and material languages I used in this remaking offers insight into a personal health history folded into bigger questions of what we might allow into an expanded field of ‘medicine’. My own lived-experience of health uncertainty is not over but I have learned that health is relational. As medieval folding almanacs demonstrate, a body can only thrive held within a flow of relationships.

## Data Availability

No datasets were generated or analysed during the current study.
